# Severe co-infection caused by difficult-to-diagnose hypermucoviscous *Klebsiella pneumoniae* K1-ST82 in a patient with COVID-19: a case report

**DOI:** 10.1186/s12879-024-10092-x

**Published:** 2024-10-28

**Authors:** Masamichi Itoga, Wataru Hayashi, Shizuo Kayama, Liansheng Yu, Yo Sugawara, Masahiko Kimura, Hiroyuki Hanada, Sadatomo Tasaka, Motoyuki Sugai

**Affiliations:** 1 Hirosaki University Graduate School of Medicine, 5 Zaihu-cho, Hirosaki, Aomori Japan; 2 Antimicrobial Resistance Research Center, National Institute of Infectious Diseases, Tokyo, Japan; 3 Hirosaki University Hospital, Aomori, Japan

**Keywords:** COVID-19, Co-infection, Hypermucoviscous *Klebsiella pneumoniae*, ST82, String test, Whole-genome sequencing

## Abstract

**Background:**

Co-infection with *Klebsiella pneumoniae* presents a significant concern in hospitalized patients with coronavirus disease (COVID-19), increasing the risk of severe disease progression. Hypervirulent (hv) and hypermucoviscous (hm) *K. pneumoniae* (Kp) has gained prominence in Asia due to its capacity to cause invasive community-acquired infections. However, recognition of hvKp/hmKp co-infections in the context of COVID-19 remains limited. We report a severe case of rapidly progressing co-infection with hmKp exhibiting “difficult-to-diagnose” phenotypes in a hospitalized patient with COVID-19.

**Case presentation:**

A 61-year-old woman with COVID-19 initially exhibited mild symptoms resembling the common cold. However, her condition rapidly deteriorated over 7 days, leading to hospital admission with the development of dyspnea. The patient required supplemental oxygen, antibiotic treatment, and mechanical ventilation. Gram-negative bacteria with atypical phenotypes were isolated from alveolar lavage fluid and blood cultures. Both strains formed small, glossy, non-lactose-fermenting colonies on clinically relevant media and were susceptible to ampicillin. Conventional biochemical tests failed to identify the *Enterobacteriales* strains owing to the urease-negative phenotype. Consequently, the identification of *K. pneumoniae* was difficult until matrix-assisted laser desorption ionization time-of-flight mass spectrometry (MALDI-TOF MS) analysis was performed. A positive string test indicated mucoviscosity, but with variability in the material used for stretching colonies. Whole-genome sequencing performed on the MiSeq and GridION platforms revealed the blood-derived strain JARB-RN-0063 as belonging to serotype K1 and sequence type (ST) 82. The hvKp-associated genes *rmpA* and *iroCD* were located on a 5.0-Mb chromosome, and *iucABCD*-*iutA* was identified on a 217.9-kb IncFIB(K)/IncR-type plasmid. Therefore, JARB-RN-0063 was genetically classified as hvKp with a Kleborate virulence score of 3. The intrinsic penicillinase gene *bla*_SHV_ was defective owing to an IS*1F* element insertion, resulting in the strain being atypically susceptible to ampicillin.

**Conclusions:**

This is the first case of severe COVID-19-associated co-infection with a difficult-to-diagnose *K. penummoniae* strain. Notably, co-infection by the hmKp K1-ST82 clone exhibited atypical phenotypes, including stunted growth, non-lactose fermentation, urease-negative reaction, ampicillin susceptibility, and abnormal mucoviscosity, posing diagnostic challenges for clinical laboratories and impedes the identification of hvKp/hmKp. Delayed identification may worsen patient outcomes, highlighting the need for increased clinical awareness of such difficult-to-diagnose clones to prevent deterioration.

**Supplementary Information:**

The online version contains supplementary material available at 10.1186/s12879-024-10092-x.

## Background

Bacterial co-infection in hospitalized patients with coronavirus disease (COVID-19) can complicate therapeutic management and increase the risk of disease severity in clinical settings. Co-infection with bacteria, particularly *Klebsiella pneumoniae* (Kp), has emerged as a significant concern in hospitalized patients with COVID-19 [1]. Approximately 55.6% of hospitalized patients infected with SARS-CoV-2 had *K. pneumoniae* co-infection during the early stages of COVID-19 [1]. Historically, *K. pneumoniae* has been a major cause of nosocomial pneumonia and urinary tract infections [2]. Notably, hypervirulent (hv)/hypermucoviscous (hm) *K. pneumoniae* has garnered attention for causing severe community-acquired infections, including pyogenic liver abscesses, bacteremia, and metastatic dissemination to distant sites, resulting in significant mortality [2, 3]. Despite the ongoing COVID-19 pandemic, studies on the co-infection of SARS-CoV-2 and hvKp/hmKp and its recognition remain limited.

Herein, we report a severe case of co-infection with a “difficult-to-diagnose” phenotype of hmKp with SARS-CoV-2 in Japan, along with the genomic characteristics of the strains.

## Case presentation

**Fig. 1:**
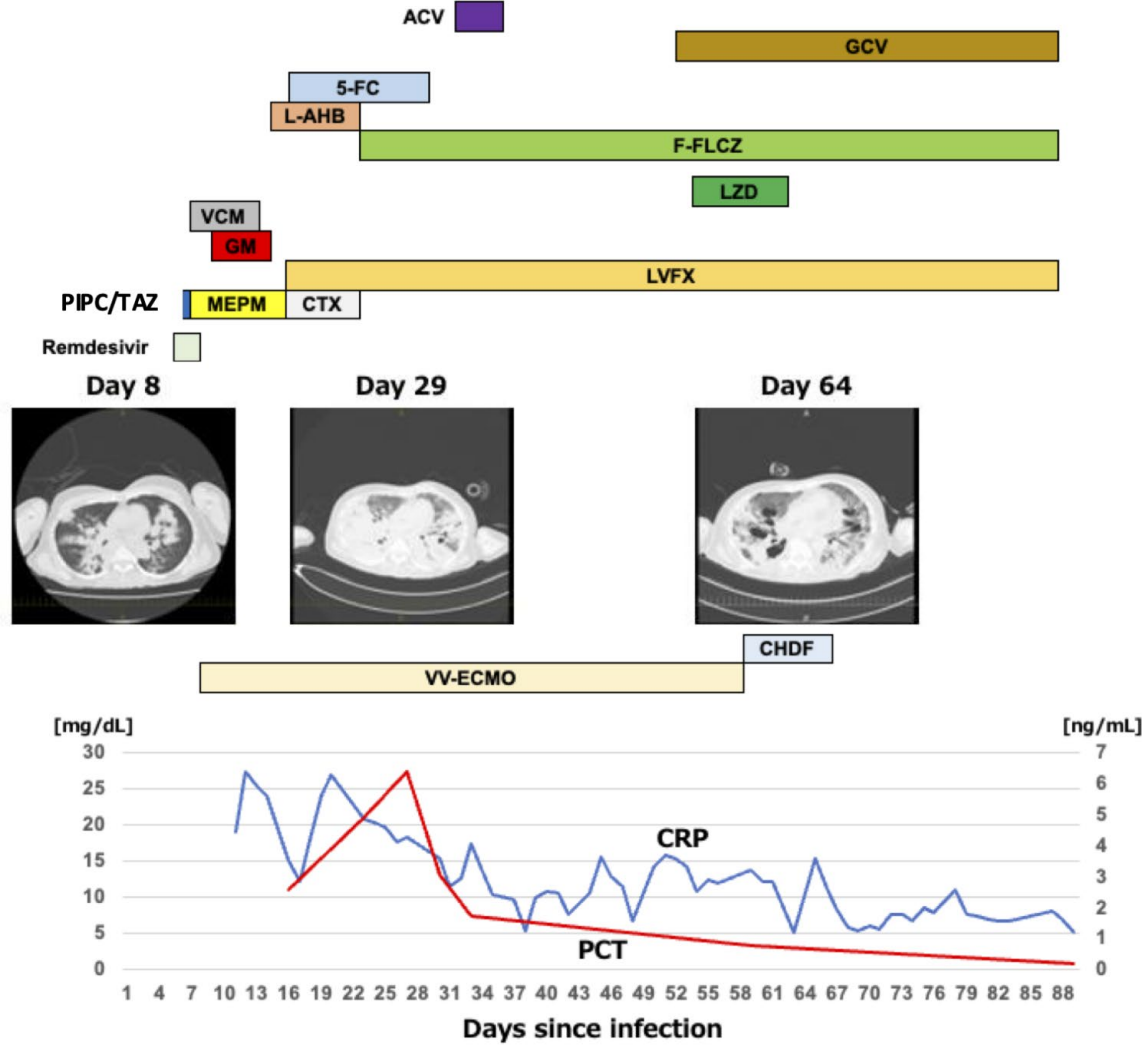
Clinical course from symptom onset. The presented data include the timeline of antimicrobial and anti-COVID-19 drug administration, introduction of VV-ECMO and CHDF, CRP, and PCT levels, and CT images. The red line indicates the PCT level, and the blue line indicates the CRP level. ACV: Aciclovir, GCV: Ganciclovir, 5-FC: 5-flucytosine, L-AHB: Liposomal amphotericin B, F-FLCZ: Fosfluconazole, LZD: Linezolid, VCM: Vancomycin, GM: Gentamicin, LVFX: Levofloxacin, PIPC/TAZ: Piperacillin/tazobactam MEPM: Meropenem, CTX: Cefotaxime, VV-ECMO: Veno-venous extracorporeal membrane oxygenation, CHDF: Continuous hemodiafiltration, CRP: C-reactive protein, PCT: Procalcitonin

In August 2022, a 61-year-old woman without any underlying health condition contracted COVID-19, initially exhibiting mild symptoms resembling the common cold. The next day, she tested positive for SARS-CoV-2 by PCR at a nearby clinic. She received symptomatic treatment (prescription of an antitussive agent, expectorant, bronchodilator, and antipyretic) and was placed under home quarantine. However, her condition deteriorated over 7 days; she developed dyspnea, requiring supplemental oxygen, and eventually required hospitalization. Despite the absence of evidence of pneumonia, the patient exhibited poor oxygenation; therefore, treatment with dexamethasone and remdesivir was initiated (Fig. 1). By day 8, a CT scan revealed pneumonia, worsening respiratory distress, and she required high-flow nasal cannula oxygen therapy. Suspecting bacterial pneumonia, Piperacillin/tazobactam was administered, and mechanical ventilation was initiated. However, her condition further deteriorated, leading to her transfer to our hospital with advanced medical care and technology 3 days after admission, where gram-negative bacteria with similar atypical phenotypes described below were isolated from alveolar lavage fluid and blood cultures. She was then diagnosed with severe pneumonia and septic shock. Despite adjusting her ventilator settings, oxygenation remained challenging; therefore, veno-venous extracorporeal membrane oxygenation (VV-ECMO) was initiated on day 10. Considering her primary conditions of severe pneumonia and sepsis, remdesivir was simultaneously discontinued. On day 18, a VV-ECMO circuit exchange was performed, and a quantitative antigen test for SARS-CoV-2, using the cobas^®^ 8000 (Roche Diagnostics) and the Elecsys SARS-CoV-2 antigen assay (Roche Diagnostics) returned negative. Chest computed tomography findings on day 19 revealed expanding lung shadows indicative of pulmonary parenchymal damage. On day 23, a tracheostomy was performed owing to the extended duration of mechanical ventilation. Throughout the disease course, antibacterial, antifungal, and antiviral agents were administered as appropriate, based on the presence of bacterial, fungal (*Candida* on day 12), or occasional viral infections (herpes labialis on day 29 and cytomegalovirus infection on day 50), respectively. Removal of VV-ECMO on day 59 helped stabilize the patient’s condition and allowed for management with mechanical ventilation. No disseminated lesions were observed. Extensive pulmonary damage indicated that weaning from mechanical ventilation is a long-term process. On day 85, the signs of infection improved, and the antibiotics were discontinued. On day 88, it was decided that no further advanced medical care was necessary, and the patient was transferred to a local general hospital. Respiratory status at the time of transfer was stable with pressure-controlled-continuous mandatory ventilation (PC-CMV), although ventilator management was required. The ventilator settings were P_I_ 18 cm H_2_O, T_I_ 1.10 s, f 22 BPM, F_I_O_2_ 0.35, and PEEP 7 cm H_2_O.

**Fig. 2:**
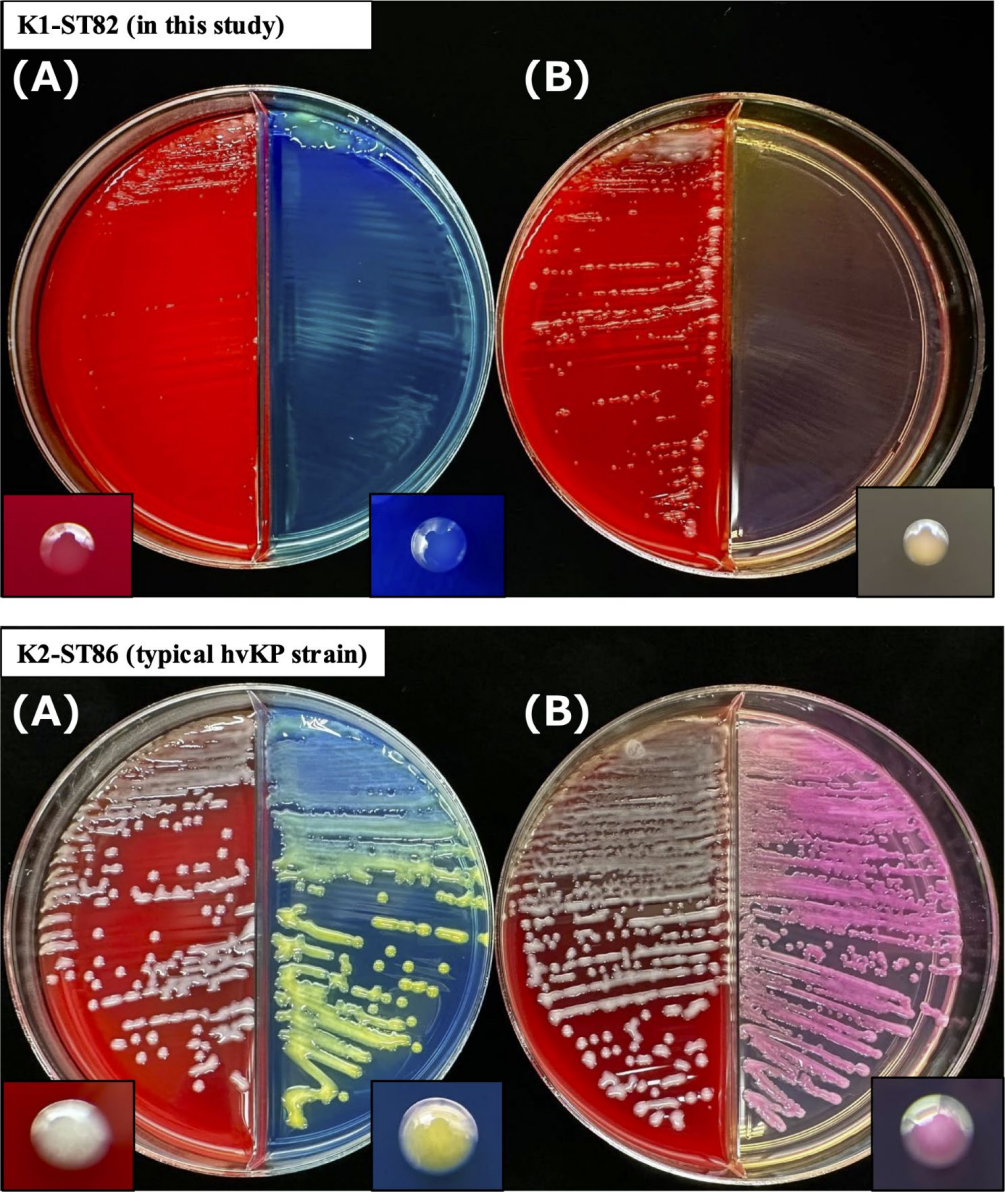
Small colonies of the K1-ST82 *K. pneumoniae* strain JARB-RN-0063 in this study and typical colonies of the K2-ST86 hypervirulent *K. pneumoniae* clinical strain JARB-RN-0064 (control) after overnight incubation at 37 °C on 5% sheep blood/Drigalski agar (**A**) and 5% sheep blood/MacConkey agar (**B**)

The strains from the two specimens exhibited atypical small glossy colonies on 5% sheep blood agar and small non-lactose-fermenting colonies with stunted growth on Drigalski and MacConkey agar (Fig. 2). In addition, the strains were atypically both susceptible to ampicillin with minimum inhibitory concentration (MIC) of ≤ 4 mg/L. Moreover, conventional identification using the ID 32 E system (bioMérieux), based on biochemical properties, could not help accurately differentiate certain *Enterobacteriales*, including *K. pneumoniae*, *Raoultella terrigena*, and *K. aerogenes*, resulting in a “low level of discrimination” owing to the urease-negative reaction (Supplemental Table S1). Consequently, identification of the strains as *K. pneumoniae* was difficult until matrix-assisted laser desorption ionization time-of-flight mass spectrometry (MALDI-TOF MS) analysis using the MALDI Biotyper sirius system (MBT Compass software version 4.1.100 and MBT-BDAL-10833 library) was performed, with a score value of ≥ 2.0. Furthermore, the strain generated a 50-mm viscous string when stretched with a wooden toothpick but did not exhibit the typical hypermucoviscous phenotype with a plastic inoculation needle, resulting in the opposite result in the string test (Supplemental Video 1, Supplemental Video 2). Therefore, this case was considered to be an infection caused by monoclonal *K. pneumoniae* because the individual strains from the two specimens had similar atypical phenotypes.

For subsequent comprehensive analyses, to characterize the genomic features of the blood-derived *K. pneumoniae* strain JARB-RN-0063, which was the only stain stored in the clinical laboratory, whole-genome sequencing (WGS) was performed using the MiSeq (Illumina) and GridION (Oxford Nanopore Technologies) platforms per the manufacturer’s instructions, followed by hybrid assemblies using the Unicycler v0.4.8 pipeline (https://github.com/rrwick/Unicycler). Multilocus sequence typing (MLST), capsular (K) serotyping, and virulence gene identification were performed using the Kleborate v2.3.1 tool (https://github.com/klebgenomics/Kleborate), and the plasmid replicon type was predicted using PlasmidFinder 2.1 (https://cge.food.dtu.dk/services/PlasmidFinder/).

WGS analysis revealed that the *K. pneumoniae* strain JARB-RN-0063 belonged to serotype K1 and sequence type (ST) 82 and possessed multiple hvKp-associated virulence genes, *rmpA* (regulator of mucoid phenotype), *iroCD* (salmochelin), and *iucABCD*-*iutA* genes (aerobactin). Therefore, this strain was genetically classified as hvKP in accordance with the presence of the genotypic biomarkers proposed by Russo et al. [4] and had a Kleborate virulence score of 3.

The *rmpA* and *iroCD* genes were located on a 5.0-Mb chromosome flanked by IS*1 × 4* elements, and the *iroCD* gene was adjacent to it (Supplemental Fig. S1). Meanwhile, the *iucABCD*-*iutA* gene cluster was identified on a 217.9-kb IncFIB(K)/IncR-type plasmid, designated as pJARB-RN-0063_1, encoding 248 open reading frames (ORFs) with a G + C content of 50.7%. A BLASTn search revealed the absence of plasmid sequences closely related to the backbone of plasmid pJARB-RN-0063_1. However, comparative analysis revealed that the complete sequence of pJARB-RN-0063_1 exhibited 25% query coverage and 99.99% nucleotide sequence identity with the IncHI1/IncFIB-type virulence plasmid pLVPK (GenBank accession no. AY378100), which is widely distributed among hvKp [2]. Additionally, pJARB-RN-0063_1 shared the genetic environment of the *iucABCD*-*iutA* operon (Supplemental Fig. S2).

Exploration of antimicrobial resistance genes revealed the absence of the penicillinase gene *bla*_SHV_, which is intrinsic to *Klebsiella* spp., suggesting an inherent susceptibility to ampicillin. A comparative analysis with the corresponding sequence of *K. pneumoniae* ATCC 35,657 (GenBank accession number CP015134) revealed that the 5.7-kb region surrounding the *bla*_SHV_ was replaced by the insertion of the insertion sequence IS*1F* element (Supplemental Fig. S3). Moreover, a lactose operon comprising the *lacI*, *lacZ*, and *lacY* genes, which are involved in lactose metabolism, was identified upstream of the IS*1F* element. However, defects were observed at the 5′ ends of the *lacZ* and *lacI* genes (Supplemental Fig. S3). We also investigated the genetic basis for the urease-negative phenotype observed in the biochemical identification test. The *ureDABCEFG* operon, which encodes the structural and regulatory components of the urease enzyme, was present on the chromosome. However, the *ureE* gene showed a single nucleotide (“A”) insertion at position + 101, causing a frameshift and premature stop codon.

## Discussion and conclusions

We presented a case of severe hmKp co-infection in a hospitalized patient with COVID-19. Typically, *K. pneumoniae* exhibits phenotypic characteristics of yellow (Drigalski agar) and pink (McConkey agar) colonies owing to lactose fermentation and inherent ampicillin resistance attributed to the penicillinase gene *bla*_SHV_, and urease activity [2, 5]. However, the hmKp strain detected in this study, in addition to being atypically susceptible to ampicillin due to the deficiency of *bla*_SHV_, exhibited atypical small glossy and non-lactose-fermenting colonies on clinically relevant media and urease-negative phenotype. These atypical phenotypic characteristics may impede the prediction and identification of *K. pneumoniae*. Prolonged incubation time of up to 48 h did not alter the appearance (colony size and non-lactose fermentation) and development of the bacterial colonies. Additionally, the growth inhibition effects of the *K. pneumoniae* strain JARB-RN-0063 using pH-indicator dyes in Drigalski and MacConkey agar were not confirmed upon their addition to the medium (data not shown). The string test, used for the phenotypic screening of hvKp, is considered positive with a viscous string > 5 mm in length from bacterial colonies on 5% sheep blood agar [2]. The difference in mucoviscosity observed in our case, depending on the material stretching the colony, may cause false negatives in the string test, implying a risk of missing hvKp/hmKp in clinical laboratories. Therefore, further analyses are required to understand the mechanisms underlying the formation of unusual small colonies and mucoviscous phenotype.

Although predominantly endemic in Asian countries, including Japan, hvKp/hmKp has recently emerged globally [2, 6, 7]. Most of these isolates belong to capsular serotypes K1 or K2, which are significant chromosomal potential virulence determinants [7, 8]. Additionally, increased virulence is attributed to the acquisition of virulence plasmids, particularly those encoding *rmpA*/*rmpA2* and siderophore genes such as *iuc* and *iro*, which characterize hvKP. Among the hvKp strains belonging to serotype K1, the ST23 clone is the most dominant, and it is strongly related to invasive liver abscesses and bloodstream infection [6]. However, the hmKp strain found in this severe case belonged to K1-ST82, which harbors multiple chromosomal/plasmid-mediated hvKp-associated virulence genes. The categorical virulence score, ranging from 0 to 5 and reflecting the clinical risk of *K. pneumoniae* infection, was 3, which is comparable to that of epidemic hvKp clones such as ST23, ST86, and ST65 [8].

A recent retrospective study in Japan revealed four cases of invasive *K. pneumoniae* infection caused by the K1-ST82 clone [9]. Genome sequence analysis of the four K1-ST82 strains (KP17009AIH, KP18003AIH, KP18062AIH, and KP19008AIH) retrieved from the GenBank database (BioProject accession number PRJDB11378) revealed that all strains exhibited hvKp-associated gene profiles (*rmpA*, *iro*, and *iuc*) and a Kleborate virulence score of 3, consistent with strain JARB-RN-0063 (Supplemental Table S2). These strains also showed genetic determinants associated with atypical phenotypes, including non-lactose fermentation, urease-negative reaction, and ampicillin susceptibility. Therefore, these atypical phenotypic characteristics and the presence of hvKp-associated virulence genes may be widespread in the K1-ST82 clone.

The pathogenic potential of the K1-ST82 clone remains poorly understood. The K1-ST82 clone exhibits fewer hvKp-associated gene profiles and a distinct genomic background compared with that of the K1-ST23 clone, suggesting lower pathogenicity in mice compared with that of other hvKp clones [9, 10]. However, the previous study did not fully elucidate the genetic background, including hvKp-associated virulence genes (*rmpA*, *iro*, and *iuc*), of the K1-ST82 clone utilized as a mouse infection model [10]. To further evaluate the pathogenicity of the K1-ST82 clone with atypical characteristics, future in vivo infection assays using mice or *Galleria mellonella* should be conducted.

A recent study showed that more than 50% of adult patients who died from COVID-19 in China experienced bacterial co-infection during hospitalization [11]. Furthermore, *K. pneumoniae* was most frequently detected in respiratory and blood samples from patients with severe COVID-19 admitted to intensive care units [12]. To the best of our knowledge, few studies have reported cases of hvKp/hmKp co-infection, predominantly involving ST23, ST86, and ST147 clones, in hospitalized patients with COVID-19 [13–16]. Thus, this is the first case of severe COVID-19-associated bacterial co-infection caused by the ST82 clone. Notably, the Japan Nosocomial Infections Surveillance (JANIS) in 2020 revealed a > 10% increase in the isolation of *K. pneumoniae* from blood samples [17]. Although co-infection with SARS-CoV-2 and *K. pneumoniae* continues to be reported [1, 18], the potential consequences of hvKp co-infection remain unclear; this highlights the need for vigilant monitoring to prevent dissemination of the K1-ST82 clone.

In conclusion, co-infection of the “difficult-to-diagnose” hmKp with atypical phenotypic characteristics poses challenges for clinical laboratories and healthcare settings during the COVID-19 pandemic, potentially leading to rapid deterioration in the clinical status of patients. Collectively, our findings highlight the critical need for clinical awareness and vigilant monitoring of the K1-ST82 clone to prevent further deterioration in hospitalized patients with COVID-19 and ensure effective infection control.

## Electronic supplementary material

Below is the link to the electronic supplementary material.

**Supplementary Material 1: Supplemental Table S1**: Biochemical characterization of the *K. pneumoniae* strain JARB-RN-0063 using bioMérieux ID 32 E identification system. The result was interpreted with the APIWEB™ identification database version 4.0 (https://apiweb.biomerieux.com/)

**Supplementary Material 2: Supplemental Table S2**: Comparison of genetic characteristics between the *K. pneumoniae* strain JARB-RN-0063 (this study) and four K1-ST82 strains (KP17009AIH, KP18003AIH, KP18062AIH, and KP19008AIH) previously reported in Japan [9]

**Supplementary Material 3: Supplemental Fig. S1**: Genetic structure of the chromosome harbored by the *K. pneumoniae* strain JARB-RN-0063 and the detailed structure of the genetic environment of the *rmpA* and *iroCD* genes are presented at the bottom. Each circle from outside to center represents the CDS/tRNA/rRNA ratio, GC content, and GC skew, and the arrows indicate predicted coding sequences (CDS). The figures were generated using the CGView server (https://cgview.ca)

**Supplementary Material 4: Supplemental Fig. S2**: Circular comparison of the complete genome sequence between IncFIB(K)/IncR-type plasmid pJARB-RN-0063_1 harbored by the *K. pneumoniae* strain JARB-RN-0063 and IncHI1/IncFIB-type virulence plasmid pLVPK (GenBank accession no. AY378100). Each circle from outside to center represents the plasmid sequence of pLVPK, CDS of pJARB-RN-0063_1, GC content, and GC skew, and the arrows indicate predicted CDS. The figures were generated using the CGView server (https://cgview.ca) and BLASTn

**Supplementary Material 5: Supplemental Fig. S3**: Linear comparison of the genetic environment surrounding *bla*_SHV_ of *K. pneumoniae* ATCC 35657 (GenBank accession number CP015134) with the corresponding region of *K. pneumoniae* JARB-RN-0063 (this study). The event of replacement by the IS*1F* element in the 5.7-kb region containing the *bla*_SHV_ and the lactose operon are highlighted, and the arrows represent the predicted CDS. The figure was generated using Easyfig software (http://mjsull.github.io/Easyfig/)

**Supplementary Material 6: Supplemental Video 1**: String test of the K1-ST82 *K. pneumoniae* strain JARB-RN-0063 using a plastic inoculation needle (left) and a wooden toothpick (right)

**Supplementary Material 7: Supplemental Video 2**: String test on the typical K2-ST86 hypervirulent *K. pneumoniae* clinical strain JARB-RN-0064 using a plastic inoculation needle (left) and a wooden toothpick (right) as a reference

## Data Availability

The datasets used and/or analyzed during the current study are available from the corresponding author upon reasonable request. The raw sequence data of the K. pneumoniae strain JARB-RN-0063 were deposited in the DDBJ Sequence Read Archive (DRA) (accession no. DRA017508).

## Abbreviations

CDS: Coding sequences
COVID-19: Coronavirus disease
DRA: DDBJ Sequence Read Archive
hmKp: Hypermucoviscous K. pneumoniae
hvKp: Hypervirulent K. pneumoniae
JANIS: Japan Nosocomial Infections Surveillance
MALDI-TOF MS: Matrix-assisted laser desorption ionization time-of-flight mass spectrometry
MIC: Minimum inhibitory concentration
MLST: Multilocus sequence typing
ST: Sequence type
VV-ECMO: Veno-venous extracorporeal membrane oxygenation
WGS: Whole-genome sequencing
