# Supplementary figures and images for: Severe co-infection caused by difficult-to-diagnose hypermucoviscous Klebsiella pneumoniae K1-ST82 in a patient with COVID-19: a case report
Source: BMC Infect Dis. 2024 Oct 28;24:1215. doi: 10.1186/s12879-024-10092-x (PMC11520518; doi:10.1186/s12879-024-10092-x)

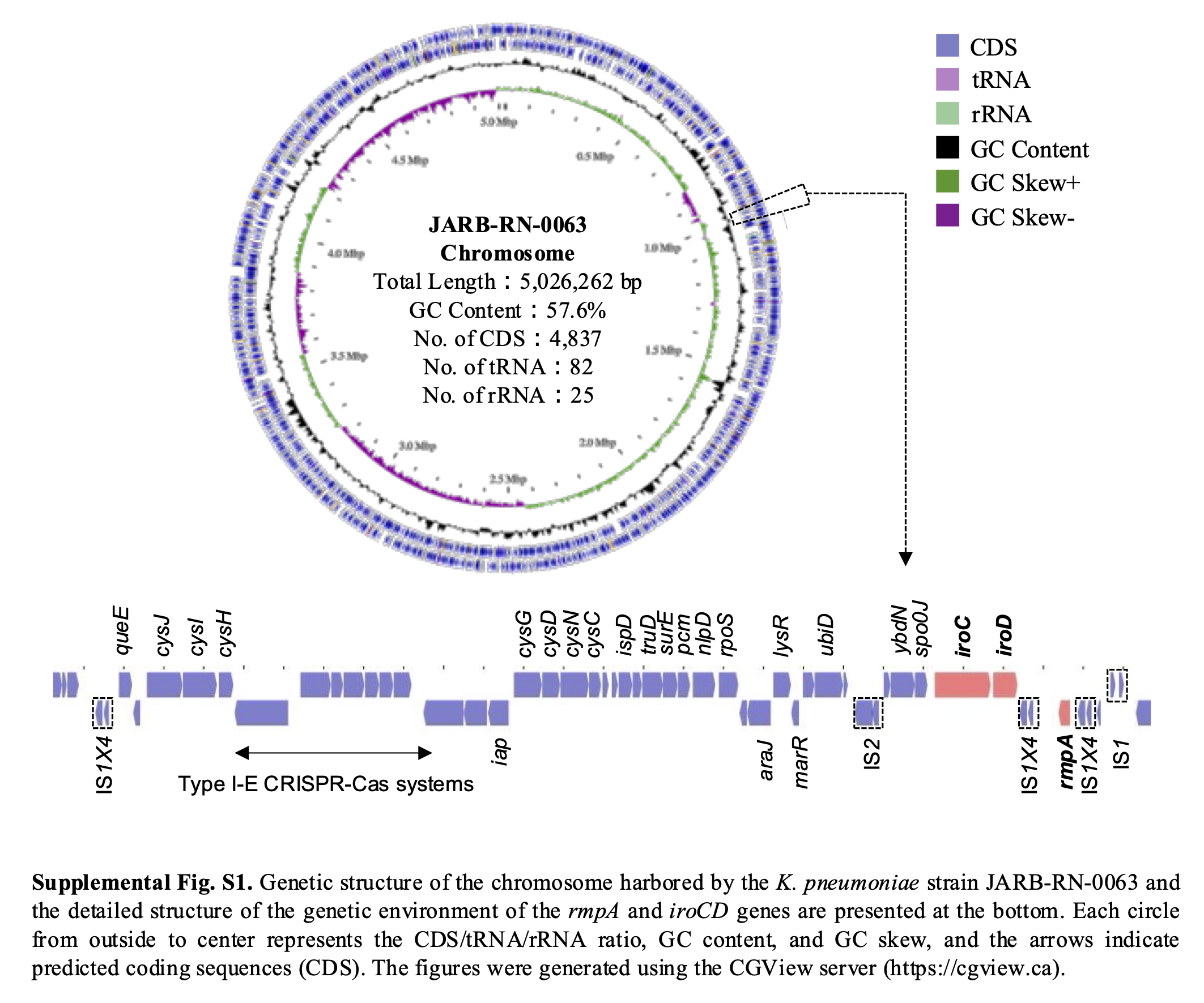

Supplement: Supplementary file 3 — Supplementary Material 3: Supplemental Fig. S1: Genetic structure of the chromosome harbored by the K. pneumoniae strain JARB-RN-0063 and the detailed structure of the genetic environment of the rmpA and iroCD genes are presented at the bottom. Each circle from outside to center represents the CDS/tRNA/rRNA ratio, GC content, and GC skew, and the arrows indicate predicted coding sequences (CDS). The figures were generated using the CGView server (https://cgview.ca) [file 12879_2024_10092_MOESM3_ESM.tiff]

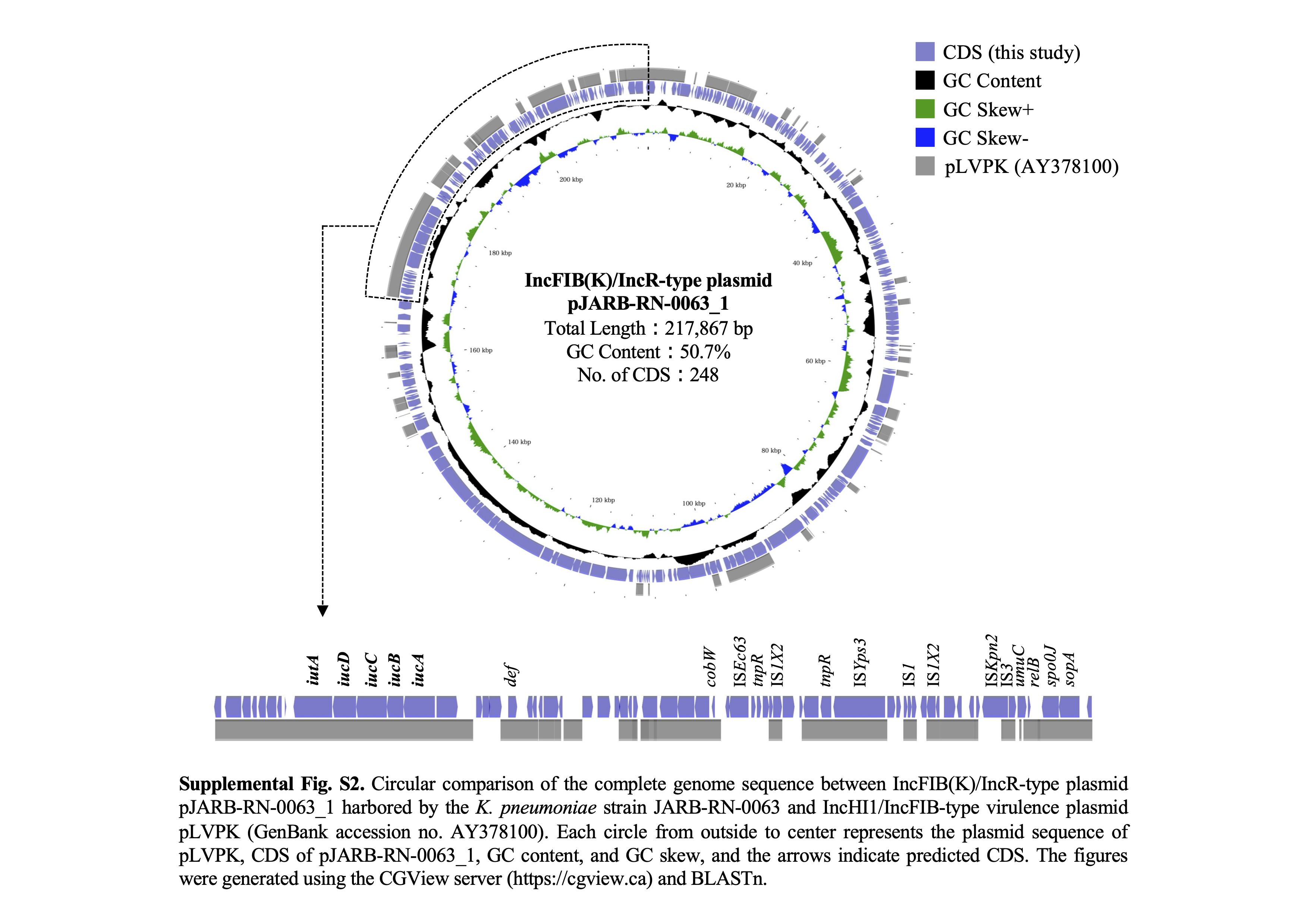

Supplement: Supplementary file 4 — Supplementary Material 4: Supplemental Fig. S2: Circular comparison of the complete genome sequence between IncFIB(K)/IncR-type plasmid pJARB-RN-0063_1 harbored by the K. pneumoniae strain JARB-RN-0063 and IncHI1/IncFIB-type virulence plasmid pLVPK (GenBank accession no. AY378100). Each circle from outside to center represents the plasmid sequence of pLVPK, CDS of pJARB-RN-0063_1, GC content, and GC skew, and the arrows indicate predicted CDS. The figures were generated using the CGView server (https://cgview.ca) and BLASTn [file 12879_2024_10092_MOESM4_ESM.tiff]

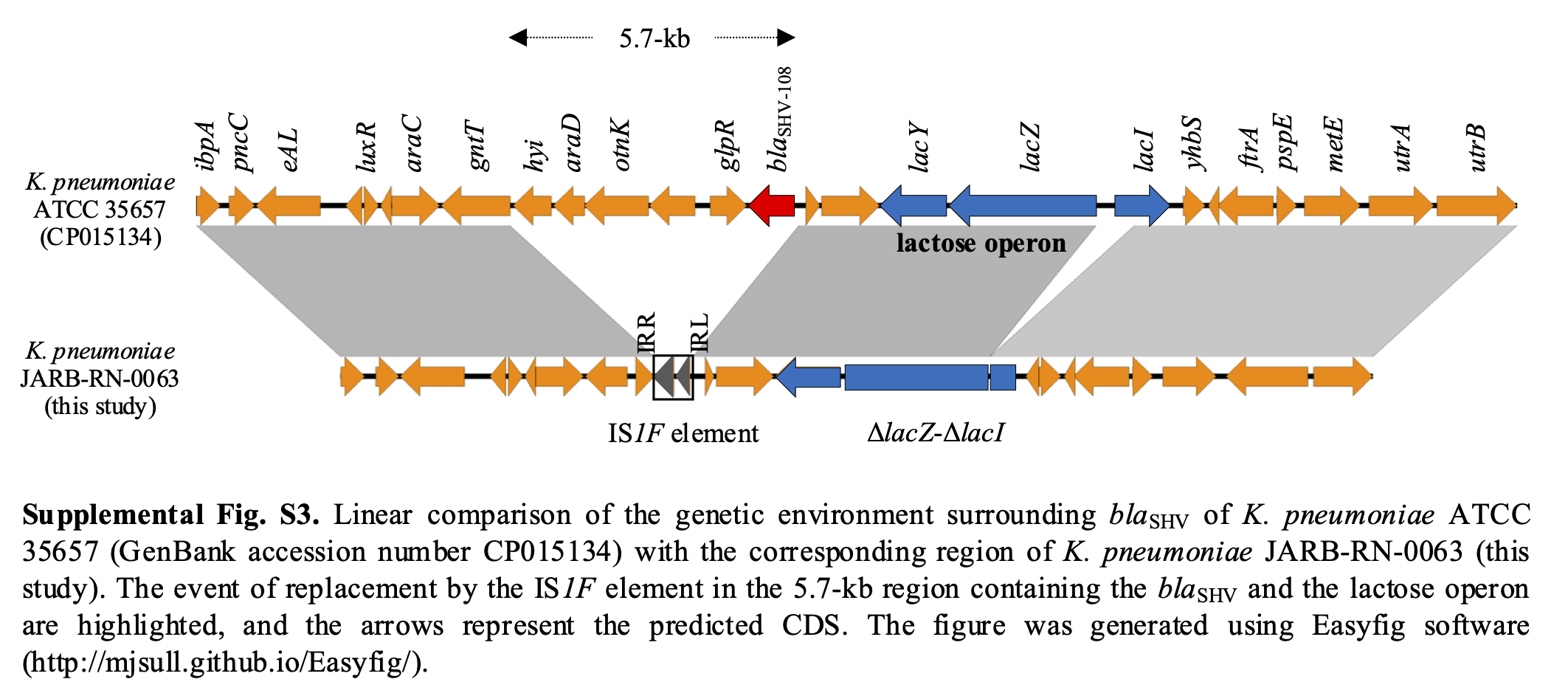

Supplement: Supplementary file 5 — Supplementary Material 5: Supplemental Fig. S3: Linear comparison of the genetic environment surrounding blaSHV of K. pneumoniae ATCC 35657 (GenBank accession number CP015134) with the corresponding region of K. pneumoniae JARB-RN-0063 (this study). The event of replacement by the IS1F element in the 5.7-kb region containing the blaSHV and the lactose operon are highlighted, and the arrows represent the predicted CDS. The figure was generated using Easyfig software (http://mjsull.github.io/Easyfig/) [file 12879_2024_10092_MOESM5_ESM.tiff]
